# American Dental Association and American Academy of Oral and Maxillofacial Radiology patient selection for dental radiography and cone-beam computed tomography

**DOI:** 10.1016/j.oooo.2025.11.013

**Published:** 2026-01-06

**Authors:** Erika Benavides, Josep R. Krecioch, Trishul Allareddy, Allison Buchanan, Martha Ann Keels, Ana Karina Mascarenhas, Mai-Ly Duong, Kelly K. O’Brien, Kathleen M. Ziegler, Ruth D. Lipman, Roger T. Connolly, Lucia Cevidanes, Kitrina Cordell, Satheesh Elangovan, Ashraf F. Fouad, Carlos González-Cabezas, Sarandeep Singh Huja, Deepak Kademani, Asma Khan, Anchal Malik, Darshanjit Pannu, Zachary S. Peacock, Mario Ramos, Hector F. Rios, Parish Sedghizadeh, Marcela Romero-Reyes, James Hawkins, Elise Watson Sarvas, Juan Yepes

**Affiliations:** a Department of Periodontics and Oral Medicine, School of Dentistry, University of Michigan, Ann Arbor, MI.; b Data Analysis and Research, Booth School of Business, University of Chicago, Chicago, IL.; c Department of Oral Pathology, Radiology and Medicine, College of Dentistry and Dental Clinics, University of Iowa, Iowa City, IA.; d Now with Department of Diagnostic Sciences, Oral and Maxillofacial Radiology, Adams School of Dentistry, University of North Carolina, Chapel Hill, NC.; e Department of Oral Health and Diagnostic Services, Dental College of Georgia, Augusta University, Augusta, GA.; f Department of Surgery, Duke University, Durham, NC.; g Department of Pediatrics, Duke University, Durham, NC.; h Department of Pediatric Dentistry, Adams School of Dentistry, University of North Carolina, Chapel Hill, NC.; i Duke Street Pediatric Dentistry, Durham, NC.; j Department of Research and Community Health, Woody L. Hunt School of Dental Medicine, Health Sciences Center, Texas Tech University, El Paso, TX.; k Now with Department of Pediatric Dentistry, College of Dentistry, Oklahoma University, Oklahoma City, OK.; l The Center for Advanced Oral Health Advanced Care Clinic, Arizona School of Dentistry and Oral Health, A.T. Still University, Mesa, AZ.; m Research Services and Scientific Information, ADA Library & Archives, American Dental Association, Chicago, IL.; n Retired from the American Dental Association, Chicago, IL.; o American Dental Association, Chicago, IL.; p Department of Orthodontics, School of Dentistry, University of Michigan, Ann Arbor, MI.; q Now with Department of Orthodontics, Adams School of Dentistry, University of North Carolina, Chapel Hill, NC.; r Department of Diagnostic Sciences, School of Dentistry, Louisiana State University, New Orleans, LA.; s Department of Oral and Maxillofacial Pathology, School of Dentistry, Louisiana State University, New Orleans, LA.; t Department of Periodontics, College of Dentistry and Dental Clinics, University of Iowa, Iowa City, IA.; u Department of Endodontics, School of Dentistry, The University of Alabama, Birmingham, AL.; v Health Information and Business Systems, School of Dentistry, The University of Alabama, Birmingham, AL.; w Department of Cariology, Restorative Sciences and Endodontics, School of Dentistry, University of Michigan, Ann Arbor, MI.; x Department of Advance Specialty Sciences, College of Dental Medicine, Medical University of South California, Charleston, SC.; y Minnesota Oral and Facial Surgery, Minneapolis, MN.; z Department of Endodontics, School of Dentistry, UT Health, San Antonio, TX.; aa Department of Restorative Dentistry and Comprehensive Care, School of Dental Medicine, University of Pittsburgh, Pittsburgh, PA.; bb Department of Restorative Sciences, Dental College of Georgia, Augusta University, Augusta, GA.; cc Department of Oral and Maxillofacial Surgery, Harvard School of Dental Medicine, Harvard University, Boston, MA.; dd Cleft and Craniofacial Clinic, Mass General Hospital for Children and Shriners Hospital for Children, Boston, MA.; ee College of Dental Medicine-Illinois, Midwestern University, Downers Grove, IL.; ff PeRios, PC Advanced Periodontics and Dental Implants, Holland, MI.; gg Department of Diagnostic Sciences, Anesthesia and Emergency Medicine, Herman Ostrow School of Dentistry, University of Southern California, Los Angeles, CA.; hh Brotman Facial Pain Clinic, Department of Neural and Pain Sciences, School of Dentistry, University of Maryland, Baltimore, MD.; ii Orofacial Pain Center, Walter Reed National Military Medical Center, Bethesda, MD.; jj Division of Pediatric Dentistry, School of Dentistry, University of Minnesota, Minneapolis, MN.; kk Department of Pediatric Dentistry, Indiana University School of Dentistry, Riley Hospital for Children, Indianapolis, IN.

## Abstract

**Background.:**

As an update to the 2012 American Dental Association and US Food and Drug Administration “Dental Radiographic Examinations: Recommendations for Patient Selection and Limiting Radiation Exposure,” this resource provides decision-making guidance on the use of various imaging modalities for general and pediatric dental care practitioners.

**Types of Studies Reviewed.:**

The American Dental Association Council on Scientific Affairs convened an expert panel of 6 members along with an expert consultant group of 18 members to develop evidence-based guidance on dental imaging. A systematic review of the literature was conducted to identify relevant systematic reviews and organizational guidelines addressing 9 clinical questions. The recommendations presented were developed by means of a non-Delphi process (ie, reaching consensus through a structured process).

**Results.:**

Due to limitations in the available evidence, consensus recommendations rather than formal guidelines were developed. A thorough evaluation of the patient history and clinical findings should precede radiographic examinations. Previously obtained images should be reviewed, and all imaging modalities, especially cone-beam computed tomography, should be used judiciously to minimize cumulative radiation exposure to the patient.

**Conclusions and Practical Implications.:**

Clinicians should base imaging decisions on the patient’s medical and dental histories, clinical examination findings, disease risk assessment, and the presence of specific clinical conditions. When used appropriately, radiographic imaging contributes to dental treatment decisions and results in optimal patient care.

In dental practice, radiographic imaging serves as an adjunct to a comprehensive clinical examination in the diagnosis and management of patients with confirmed or suspected dental disease, pathosis, injury, or other clinical signs or symptoms (eg, pain or trauma). The rapid advances in imaging modalities in the past several decades has led to an increased use of digital imaging, portable hand-held radiography systems, and 3-dimensional (3D) cone-beam computed tomography (CBCT).^1^ In routine dental settings, dentists encounter manifestations that require comprehensive clinical examination of each patient, including assessment of clinical signs, symptoms, and relevant patient history to determine whether diagnostic imaging is necessary to support accurate diagnosis, effective treatment planning, and appropriate patient management.

Our consensus statement provides evidence-based recommendations for general and pediatric dentists on appropriate clinical indications for patient selection, timing and frequency of diagnostic imaging procedures, and the recommended use of imaging modalities, including 2-dimensional (2D) radiographs and CBCT. This statement is not a substitute for the clinical judgment of the dental care provider. These recommendations do not provide any assurance or guarantee of specific patient outcomes and are subject to revision as new evidence emerges that warrants further evidence-based review or updates to the guidance presented herein.

This consensus statement is the second of a 2-article series addressing dental imaging guidance for clinical indications. The first article, published in 2024, presented recommendations on radiation safety, appropriate imaging practices, and regulatory oversight.^2^

Dentists should comply with this patient selection update, the 2024 safety guidance,^2^ and the 2012 American Dental Association (ADA) and US Food and Drug Administration (FDA) recommendations when prescribing dental radiographs^3^ and follow the as low as reasonably achievable and as low as diagnostically acceptable principles. Imaging must be justified by means of the clinical examination, dental and medical histories, and patient’s disease risk assessment. Consider the patient’s age, dental development, and disease risk, and prioritize clinical benefit over routine or convenience-based use. Clinicians should reserve CBCT for justified clinical indications, refer to specialists when appropriate, and avoid duplicate imaging.

## METHODS

### Expert Panel and Consultant Group

The ADA Council on Scientific Affairs (CSA) agreed to develop evidence-based recommendations on dental radiography and images obtained with CBCT as an update to the 2012 ADA and FDA “Dental Radiographic Examinations: Recommendations for Patient Selection and Limiting Radiation Exposure”^3^ and the 2012 advisory statement on the use of CBCT in dentistry.^4^ To oversee this initiative, an expert panel was formed, comprising general, public health, and pediatric dentists and oral and maxillofacial radiologists (E.B., T.A., A.B., M.A.K., A.K.M., M.L.-D.). An oral and maxillofacial radiologist (E.B.) was nominated and approved by the ADA CSA to serve as chair of the expert panel.

A consultant group of dental specialty provider types was formed to provide subject matter expertise. The following provider types were represented: cariology (C.G.-C., A.M.), endodontics (A.F.F., A.K.), oral and maxillofacial pathology (K.C., P.S.), oral and maxillofacial surgery (D.K., Z.S.P.), orthodontics (L.C., S.S. H.), orofacial pain (M.R.-R., J.H.), pediatric dentistry (E.W.S., J.Y.), periodontics (S.E., H.F.R.), and prosthodontics (D.P., M.R.). The ADA CSA provided administrative oversight for the project, with additional support from ADA staff members.

The expert panel served as the voting group for the recommendations presented. The consultant group contributed subject matter expertise and offered diverse perspectives on provider type—specific imaging considerations for review, discussion, and voting. All members of the expert panel and consultants provided disclosures of potential conflicts of interest, which were reviewed and updated periodically throughout the project. None of the expert panelists or consultants disclosed conflicts that precluded participation in the project.

### Search strategy and literature review

An informationist (K.K.O.) developed a comprehensive search strategy to identify systematic reviews and national and international clinical practice guidelines related to dental imaging modalities. The search focused on 2D radiographs (including bite-wing, periapical, occlusal, panoramic, and cephalometric) and 3D imaging (CBCT), as used in general dentistry and among recognized dental specialty provider types, with a primary focus on digital imaging modalities. The full list of search strategies is provided in Appendix 1 (available online), and a flow diagram is shown in the eFigure (available online).

A search strategy was developed to identify literature related to indications and recall visit frequency for dental imaging. The Scottish Intercollegiate Guidelines Network systematic review filter was modified to include guideline language and applied to searches in MEDLINE and Embase.^5^

The search was conducted in August 2020 in Ovid MEDLINE 1946 to present, Embase 1947 to present, and the Cochrane Database of Systematic Reviews, and was updated in October 2024. Database-supplied limits were used to restrict items to those published in the past 10 years. No language limits were applied. The quality of the identified guidelines was evaluated using the Appraisal of Guidelines for Research and Evaluation II tool.^6^ Evidence syntheses derived from the included guidelines and systematic reviews were reviewed by the primary expert panel. In addition, consultants were invited to recommend supplementary literature outside the scope of the primary search strategy, including nonsystematic reviews or laboratory-based studies on the basis of their contextual relevance.

### Selection of clinical questions

During the initial project meetings, the expert panel and consultants developed a set of 9 key clinical questions, which were unanimously approved by the expert panel. These clinical questions served as the overarching scope for the development of the recommendations presented within our consensus statement. The full list of clinical questions is provided in Appendix 2 (available online).

### Development of recommendations

The library search results and evidence summaries prepared by ADA staff members were used by the consultants to draft provider type—specific recommendations. Using a non-Delphi consensus process (ie, reaching consensus through a structured process), the expert panel reviewed and modified these draft recommendations before returning them to the consultants for final approval. Boxes 1 through 3 and Tables 1 and 2 containing the recommendations were compiled and jointly approved by both the consultants and expert panel members.

External review of our report was performed by members of the following organizations: the American Academy of Cariology, American Association of Endodontists, Academy of General Dentistry, American Academy of Oral and Maxillofacial Pathology, American Academy of Oral and Maxillofacial Radiology, American Association of Oral and Maxillofacial Surgeons, American Academy of Orofacial Pain, American Academy of Orthodontists, American Academy of Pediatric Dentistry, American Academy of Periodontology, and American College of Prosthodontics. All comments from these external organizations were reviewed in collaboration with the chair of the expert panel and revisions were incorporated when appropriate.

## RESULTS

The initial and updated searches identified 1,839 articles related to imaging indications and recall visit frequency. After removing duplicates and conducting abstract and full-text screening, a total of 60 articles were included for review. Those 60 articles subsequently underwent data extraction, with information recorded in either an Excel (Microsoft Corp) database or in Word (Microsoft Corp) files.

The quality of existing clinical practice guidelines on dental radiographs and images obtained using CBCT was evaluated using the Appraisal of Guidelines for Research and Evaluation II tool.^6^ Mean domain scores ranged from 9.7 through 26.4 and were all below the level typically required for guideline recommendation.^62^ Due to concerns about the level of available evidence, the expert panel decided to develop consensus recommendations rather than a formal clinical guideline.

Our recommendations were informed by means of the evidence summarized from the identified systematic reviews and existing guidelines in oral and maxillofacial imaging, along with subject matter expert input from the consultant group. The expert panel held multiple online meetings over a 4-year period (2021–2025) with the aim of reaching consensus in developing these recommendations on clinical indications and patient selection. The recommendations in our consensus statement were also developed to be aligned and consistent with the expert panel’s earlier article on clinical recommendations for optimizing radiation safety in dentistry.^2^

### Clinical recommendations

#### General Recommendations for Dental Imaging.

Consistent with both the 2012 ADA and FDA “Dental Radiographic Examinations: Recommendations for Patient Selection and Limiting Radiation Exposure”^3^ and the 2024 “Optimizing Radiation Safety In Dentistry: Clinical Recommendations and Regulatory Considerations,”^2^ the recommendations developed by the expert panel were aimed at balancing the minimization of radiation exposure with the need to obtain adequate diagnostic information. Factors such as patient age and stage of dental development are among the considerations factored into decision making regarding appropriate imaging recommendations.

Box 1 contains recommendations on adherence to the guidance on patient radiation protection, including compliance with all applicable federal, state, and local regulations; public health guidance; and manufacturer’s instructions regarding equipment optimization and use, quality assurance, and quality control as well as education and training.^1,2,4,7,63^ Box 1 contains best practices consistent with the principles of using doses as low as reasonably achievable and as low as diagnostically achievable. These include recommendations on dose optimization, diagnostic technique, quality assurance, and effective communication with patients about the risks and benefits associated with dental radiographs and CBCT. The dentist who orders or uses CBCT is responsible for interpreting it, which includes all findings within the scanned volume, even those outside the primary area of interest. This is a professional and legal requirement, and the standard of care for interpretation applies whether they specialize in radiology or not. For complex cases or a lack of expertise, referring the scan to a specialist in oral and maxillofacial radiology is a recommended means to provide higher-level patient care and mitigate the risk of missing clinically relevant incidental findings.

#### Recommendations for Specific Clinical Indications.

Box 2 contains a compilation of imaging recommendations for specific clinical indications and pathoses commonly seen and, when not referred to a dental specialty provider type, treated in a general dental practice.

Box 2, section 1, contains imaging recommendations for caries indications and includes specific recommendations for anterior proximal (Box 2, section 1.2), posterior proximal (Box 2, sections 1.3 and 1.4),^3,12^ and occlusal surfaces (Box 2, section 1.5)^3,12^ as well as for root (Box 2, section 1.6)^3,12,16^ and smooth surfaces (Box 2, section 1.7). Decision making about radiographic use, including the selection of bite-wing or periapical radiographs, should consider the lesion surface, the anatomic considerations (ie, whether the anterior or posterior proximal spaces are closed), and clinical judgment. For teeth with smooth surface caries and no clinical signs or symptoms associated with pulpitis or apical periodontitis, visual examination is preferred over radiographic imaging (Box 2, section 1.7). There is a lack of evidence to support the use of CBCT for the detection of caries (Box 2).^10,13–15^

Box 2, section 2, contains imaging recommendations for periodontal disease.^3^ The section begins with recommending the frequency of radiographic examinations should be guided by means of clinical findings and treatment response (Box 2, section 2.1).^3,17^ Radiographic evaluation of disease sites before treatment is important for diagnosis and determination of disease baseline (Box 2, section 2.2).^17^ However, the presence and severity of radiographic bone loss is not an indicator of active disease (Box 2, section 2.3).^17^ Clinical examination, along with a 2D full-mouth radiographic series, including vertical bite-wing radiographs as necessary, is recommended for evaluating periodontal disease (Box 2, section 2.4).^17^ There is no evidence to support the use of CBCT in the management of periodontal disease, except for treatment planning of complex cases (Box 2, section 2.5).^3,17,64^ For patients with history of, or with, active periodontal disease, vertical bite-wing radiographs are recommended to assess bone levels in the permanent dentition (Box 2, section 2.6).^17^

Box 2, section 3, contains imaging recommendations for orthodontic and dentofacial development indications. It begins with recommending panoramic radiography as the initial imaging modality for monitoring tooth eruption and dentofacial development before orthodontic treatment and for the assessment of root alignment during treatment (Box 2, section 3.1).^18–20^ A lateral cephalogram may be indicated, depending on the severity of the malocclusion and to assess dentofacial development (Box 2, section 3.2).^18–21^ Periapical radiographs are recommended for assessing root morphology, blunting, resorption, and the presence of periapical lesions (Box 2, section 3.3).^18–23^ For the assessment of facial asymmetry, low-dose CBCT is the recommended imaging modality. When low-dose CBCT is not available, a posteroanterior cephalometric radiograph may be used as an alternative (Box 2, section 3.4).^19,20,24^ When planning interradicular mini-implant placement, images obtained using CBCT can aid in optimal site selection and have been found to improve the mini-implant success rate compared with the use of 2D radiographs alone.^20,25^ However, the potential benefits of CBCT should be weighed against the increased radiation dose on a case-by-case basis (Box 2, section 3.5).^20,25^ When clinical indications exist (eg suspected pathology, delayed eruption, or orthodontic concerns), panoramic radiography is recommended for assessment and treatment planning.^26–32^ However, repeated panoramic imaging should be avoided unless justified by means of considerable changes in clinical status or developmental stage (Box 2, section 3.6).^3,25,65,66^

Box 2, section 4, contains recommendations for the evaluation of third molars, supernumerary teeth, and supplemental teeth, including insight framing decision making about when CBCT might be considered. Routine radiographic screening for third molars, supernumerary teeth, and supplemental teeth, in the absence of clinical indications, is not recommended (Box 2, section 4.1).^3,20^ When there is a clinical indication for radiographic evaluation of third molars, supernumerary teeth, and supplemental teeth, panoramic radiography is recommended for assessment and treatment planning (Box 2, section 4.2).^26–33^ The frequency of panoramic radiography for third molars, supernumerary teeth, and supplemental teeth should be determined on the basis of the stage of dental development and clinical need (Box 2, section 4.3).^3,26–29,33^ The frequency of imaging should be determined on the basis of patient need and not insurance coverage or convenience.

When panoramic radiography indicates an increased risk of developing an inferior alveolar nerve injury, such as root apexes located below the canal, there is darkening of the roots, loss of the cortical outline of the canal, or diversion of the canal, CBCT should be considered (Box 2, section 4.4).^30,31,33^ CBCT should only be used if the radiographic findings obtained will affect treatment decision making or risk assessment (Box 2, section 4.5).^3,4,29–32^

Box 2, section 5, contains recommendations regarding radiographic evaluation of dental patients with head and neck lesions. It is recommended that the prescription, type, and frequency of radiographic examinations for such pathoses should be determined on the basis of the patient’s history, clinical findings, potential diagnoses, and existing imaging (Box 2, section 5.1).^2,3^ An individualized approach is advised for selecting the most appropriate imaging modality for evaluating jaw lesions, given the broad spectrum of diseases (Box 2, section 5.2).^2,3,34^ Depending on clinical parameters, including manifestation, history, symptomatology, examination findings, laboratory or diagnostic test results, and therapeutics considerations, dental care provider types such as oral and maxillofacial surgery, oral and maxillofacial radiology, oral and maxillofacial pathology, oral medicine, and orofacial pain may use a variety of imaging modalities. These include, but are not limited to, 2D radiographs, CBCT, multidetector computed tomography (MDCT), positron emission tomography, magnetic resonance imaging (MRI), magnetic resonance angiography, and ultrasonography (Box 2, section 5.3). If pathosis is suspected or identified on radiographic imaging, referral to an appropriate specialist may be warranted (Box 2, section 5.4).

Box 2, section 6, contains recommendations regarding imaging of patients with temporomandibular disorders and orofacial pain. Panoramic radiography may be considered initially to identify gross osseus abnormalities but may not be sufficient for definitive diagnosis (Box 2, section 6.1).^35^ CBCT is recommended for evaluating the bony components of the temporomandibular joint, including degenerative joint disease, systemic arthritides, and condylar anomalies (Box 2, section 6.2). MDCT or CBCT is preferred when trauma or suspected condylar fractures or ankyloses are present, and MRI is preferred for detection of adhesions and for the detection of hemarthrosis (Box 2, section 6.3).^35–39^ MRI remains the reference standard for evaluating temporomandibular joint soft-tissue abnormalities, including disk displacement, disk perforations, joint effusion, and inflammation (Box 2, section 6.4), and is best performed with closed-mouth and open-mouth views when functional internal derangements are suspected (Box 2, section 6.5).^35,40^ Imaging should not be routine but reserved for cases when clinical findings indicate the need for further evaluation or treatment planning (Box 2, section 6.6).^39,41^ In cases when orofacial pain is suspected to arise from nonodontogenic and non—temporomandibular disorder causes such as neuropathic, neurovascular, neoplastic, or systemic conditions, appropriate referral to specialty provider types and advanced imaging (eg, MRI, MDCT, or positron emission tomography) are recommended to guide diagnosis and management (Box 2, section 6.7).^39,42^

Box 2, section 7, contains imaging recommendations for dental implant therapy from initial consultation through surgical placement, restoration, and maintenance. This section contains specific recommendations about the use of CBCT in implant therapy. Although panoramic radiography may be used for initial assessment before dental implant placement, 3D assessment with CBCT is recommended for presurgical planning of dental implants (Box 2, section 7.1.1).^43–45^ Recommended applications of 3D imaging in implant therapy are case-dependent and include the following applications: assessing implant sites when the clinical examination indicates the need for bone grafting or bone reconstruction (Box 2, section 7.2.1),^43,44^ assessing bone volume at edentulous sites planned for dental implant placement (Box 2, section 7.2.3),^43–45^ assessing the maxillary sinus and alveolar ridge before augmentation procedures (Box 2, section 7.2.4),^43–45^ assessing autogenous bone donor sites (Box 2, section 7.2.5),^43–45^ planning for the fabrication of static surgical guides or use of dynamic navigation during implant placement (Box 2, section 7.2.6),^43–45^ assessing previously augmented sites before implant placement (Box 2, section 7.2.7),^43–45^ and assessing complications associated with previously placed implants or grafting (Box 2, section 7.2.8).^43–46^

At the time of implant restoration delivery, a 2D intraoral radiograph, such as a bite-wing, should be obtained to establish baseline peri-implant bone levels for long-term radiographic assessment (Box 2, section 7.3.1).^45^ During implant maintenance, the need for 2D radiographic evaluation should be determined by means of clinical judgment (Box 2, section 7.4.1).^3,43,45^ To assess peri-implant bone, 2D intraoral radiography is recommended (Box 2, section 7.4.2).^43,45^ However, to assess complications, such as improper anatomic location of the implant, CBCT is recommended (Box 2, section 7.4.3).^43,45,46^

Box 2, section 8, contains imaging recommendations for tooth autotransplantation. CBCT is recommended to assess both the integrity of the donor tooth and the recipient site (Box 2, section 8.1).^48–50^ In addition, CBCT is useful in the fabrication of a donor tooth replica to be used for try-in (Box 2, section 8.2).^48–50^ However, to assess the survival of the autotransplanted tooth, 2D imaging is recommended (Box 2, section 8.3).^3^

#### Recommendations for Endodontics.

Table 1 contains recommendations for endodontic applications. Although CBCT may not be needed for every endodontic diagnosis or treatment, the indications listed are aimed at providing clinicians with appropriate concepts to keep in mind for decision making about the use of CBCT. One of these is the potential limitations of periapical radiographs. Structures within the oral cavity, including restorative materials, can introduce artifacts on periapical radiographs. In addition, there are times when it can be difficult for clinicians to see relevant structures, anatomy, or pathoses clearly. All teeth other than maxillary anterior teeth have roots with a 10% through 70% chance of additional canals in the same root that are in the buccolingual plane. There are also instances in which periapical radiographs are inadequate for detecting endodontic pathosis or identifying potential problems. In such cases, CBCT and 2D imaging modalities can provide valuable complementary information. The determination to use CBCT should be case-specific and based on clinical judgment. When additional diagnostic information is needed, CBCT is commonly the imaging modality of choice.

A comprehensive clinical examination along with appropriate radiographic imaging^8^ is integral for the diagnosis of endodontic pathosis and effective treatment planning.^2^ It is estimated that more than 80% of endodontists in the United States use CBCT in their practice, and this percentage is likely growing.^54,67,68^ CBCT should be considered if the additional information is likely to aid in diagnosis and treatment planning or enhance clinical management, particularly when 2D radiography is inconclusive.^51^

CBCT should be considered in the following clinical scenarios

- Clinical signs and symptoms and other diagnostic imaging are inconclusive
- Teeth with a history of traumatic dental injuries
- Suspicion of horizontal or longitudinal cracks or fractures
- Suspicion or evidence of root resorptive defects, including cervical, inflammatory, replacement, or internal root resorption
- Suspicion of maxillary sinusitis of endodontic origin
- Suspicion or evidence of nonendodontic pathoses that mimics endodontic disease
- Planning root canal treatment on teeth with suspected additional canals that are obscured because of overlapping on 2D images
- Management of teeth with secondary, persistent, or recurrent endodontic disease
- Presence of congenital dental anomalies, such as dens in dente or palatal groove defect
- Procedural mishaps, when enhanced imaging would facilitate management
- Midtreatment in calcified cases when root canals cannot be located with conventional methods
- Preoperative planning in surgical endodontic cases or in surgical or nonsurgical cases when guided technologies will be used
- Inability to obtain intraoral radiographs, such as in cases of trismus, severe trauma, or for patients with disabilities
- In cases of uncertainty, for definitive endodontic posttreatment evaluation, especially after surgical endodontic treatment

#### Recommendations for Pediatric Patients.

Box 3, section 1, contains general considerations for radiographic imaging in pediatric patients and dentofacial development. Radiographic imaging should be justified and obtained only after clinical examination, review of patient medical and dental histories, and risk assessment of caries and periodontal disease, and trauma (Box 3, section 1). Recommendations are provided for the frequency of radiographic examinations (Box 3, section 1.1), including initial (Box 3, section 1.1),^3,8,11,27^ and follow-up examinations (Box 3, section 1.2).^3,8,11,27^ Considering the susceptibility of children and young adults to the effects of radiation, radiographs should be prescribed judiciously, and effective dose-reduction methods (eg, selection criteria, collimation, and optimization of exposure settings) should be used (Box 3, section 1.4).^2,3,8,61^ With the use of such methods, radiation doses from dentomaxillofacial imaging carry negligible risk, therefore, routine use of lead shielding for pediatric patients is not indicated (Box 3, section 1.5).^2,7^

For pediatric patients with primary, mixed, or early adolescent dentition who have severe gingival inflammation resulting from a systemic condition, it is recommended that the initial assessment include maxillary, mandibular occlusal and periapical, and bite-wing radiographs. In these cases, tooth mobility should be evaluated in lieu of periodontal probing of hemorrhagic gingiva (Box 3, section 2.1).^3,27^

For extraoral radiographs, extraoral bite-wing raadiographs should be obtained judiciously because they deliver approximately 3 times the radiation dose found in intraoral bite-wing radiographs and may have lower diagnostic quality. Their use may be appropriate for children with special health care needs or those who are unable to tolerate intraoral radiography. They should only be considered after a clinical examination and assessment for caries and periodontal disease risk, trauma experience, eruption deviations, and third molars.^2,3,12^

#### Recommendations for Imaging Frequency.

Table 2 contains recommendations for imaging frequencies for both new and recall patient visits in the diagnosis and monitoring of caries and periodontal disease. It recapitulates the information from the 2012 ADA and FDA “Dental Radiographic Examinations: Recommendations for Patient Selection and Limiting Radiation Exposure.”^3^ Consistent with the recommendations from Box 1, section 1.7, and Box 3, section 4.1, the available evidence does not support the use of CBCT for caries detection.

## DISCUSSION

The recommendations we presented in this consensus statement provide clinicians with guidance for decision making about the use of 2D radiographs and 3D imaging in clinical practice. Imaging decisions should be patient-centered and made on the basis of the clinical question to be addressed. Dental radiographs are the most common radiologic procedure performed in the United States, with an estimated 320 million radiographs (including scans obtained by means of CBCT) completed in 2016, but the total effective dose is minimal compared with that of certain medical imaging procedures.^69^ Careful patient selection and compliance with the guidance provided in the 2024 “Optimizing Radiation Safety in Dentistry: Clinical Recommendations and Regulatory Considerations” are essential to minimize unnecessary patient radiation exposure.^2^

The clinician’s decision to obtain a radiograph or use CBCT should be made on the basis of the patient’s medical and dental history, findings from the clinical examination, disease risk assessment, and the presence of specific clinical conditions. As part of this decision-making process, clinicians should retrieve and review previously obtained radiographic images, both from their own records and from other clinicians, whenever available. The benefits of obtaining radiographic imaging to the diagnosis and treatment planning must outweigh the potential risks associated with radiation exposure.

These recommendations are intended to serve as a resource for the dental care practitioner and are not intended as standards of care, requirements, or regulations. These consensus recommendations highlight the need for more rigorous primary research on this topic.

The provider type—specific recommendations included in this consensus statement were developed by the specialty consultants and are specifically tailored to address imaging questions encountered by general dentists. When a specialist referral is warranted, general dentists should refrain from obtaining additional images that might be better obtained by the specialist to ensure diagnostic value and reduce unnecessary radiation exposure.

Developing technology relevant to imaging decisions may include artificial or augmented intelligence (AI).^70^ In a 2022 review article, researchers found AI applicable in dental radiography for image quality enhancement, diagnostic support, treatment planning, and tooth and dental implant system recognition.^9^ However, clinical use of AI in clinical practice remains limited, with the need for the development of standardized performance metrics and enhanced data set quality to improve reproducibility, mitigate biases, and support outcomes relevant to patient care. The first US standard on the use of AI in dentistry was finalized and published in early 2025, coinciding with the completion of this consensus statement’s development.^71^

## CONCLUSIONS

This consensus statement contains specific clinical recommendations for patient selection and updates the 2012 ADA and FDA recommendations^3^ regarding patient selection, indications, and recall visit frequency, which complement the expert panel’s 2024 article on patient protection and radiation dose optimization.^2^

The clinical recommendations provided in this consensus statement are intended to serve as a framework to support the professional judgment of general and pediatric dentists. Imaging decisions should be made on the basis of comprehensive clinical examination along with a thorough review of the patient’s medical, dental, and clinical histories. Radiographic exposure should be justified when the potential diagnostic or treatment benefit outweighs the associated risks. Insofar as these recommendations are directed toward the general dentist, recommendations for dental specialty provider types are beyond the scope of this article. Dental specialty organizations are encouraged to develop their own evidence-based recommendations tailored to their respective areas of expertise.

## Supplementary Material

Appendix A and B

efigure

Supplementary material associated with this article can be found in the online version at doi:10.1016/j.oooo.2025.11.013.

## Figures and Tables

**Table 1.** Endodontics recommendations.

STAGE AND INDICATION	RADIOGRAPHIC RECOMMENDATIONS	ADDITIONAL GUIDANCE
**Initial Assessment**		
Tooth evaluation	Intraoral 2D* radiographs should be considered the primary imaging modality of choice.^8,51^ CBCT^†^ may be indicated after an initial clinical examination^‡^ and assessment of 2D imaging.^8^ CBCT should be considered for patients with contradictory or nonspecific clinical signs and symptoms, unusual root or apical anatomy, suspected but unclear apical pathosis, and large apical radiolucencies involving multiple teeth, as well those involving 1 or both cortical plates, calcified canals, root resorption, external cervical resorption, previously endodontically treated teeth (including cases with persistent postoperative pain), and those with suspected perforations or separated instruments.^8,51^ CBCT should also be considered when maxillary sinusitis of endodontic origin is suspected.^52^	If CBCT is indicated, the smallest field of view consistent with the needed information should be used. Using lower voxel sizes of ≤ 0.1 mm was found to improve diagnostic accuracy in certain situations.^53^
**Diagnosis and Treatment Planning**		
Diagnosis and management of dentoalveolar trauma	CBCT is the imaging modality of choice in patients with confirmed or suspected dentoalveolar trauma (which may not be adequately assessed with 2D radiographs).^51^	Other advanced imaging modalities, such as magnetic resonance imaging, ultrasonography, or sialography may be indicated for salivary gland or other soft-tissue injury: “… in the absence of other maxillofacial or soft issue injury that may require other advanced imaging modalities.”^8^
Treatment planning
Suspected root canal anomalies	CBCT is indicated for treatment of teeth with potential for extra canals and suspected complex morphology.^8,51^	CBCT provides enhanced detection of periapical pathosis and eliminates the distortion and superimposition of bony and dental structures seen on periapical radiographs.^54^ In addition, axial plane CBCT images aid in determining the centrality of the canal within the root, the location, branching and convergence of root canals within the root, and the location of the apical foramen or foramina.
Management of endodontically treated teeth with secondary, persistent, or recurrent disease	CBCT is indicated for assessment of possible causes of new, persistent, or recurrent apical periodontitis, determining indications and strategies for surgical or nonsurgical retreatment^2^ (eg, large periapical lesions in posterior teeth, and the evaluation of their proximity to adjacent relevant anatomic structures).^8,51,55,56^ CBCT also enables determination of the size of the lesion, and the involvement of the buccal or lingual cortical plates. This assists in surgical planning and determining the need for grafting.^57,58^	Initial screening for these cases is frequently done by a general dentist, who should be able to discuss options of further treatment with the patient and consult with the endodontist accordingly. A cone-beam computed tomographic scan may also assist when discussing both endodontic and nonendodontic options for the patient.
Intentional reimplantation and autotransplantation	CBCT is indicated for intentional reimplantation and autotransplantation.	CBCT provides 3-dimensional confirmation of the compatibility of root anatomy with atraumatic extraction. It is also needed for the fabrication of a 3-dimensional tooth replica of the donor tooth.^48–50^
**CBCT to Assist With Difficult Treatment**		
Intra-appointment imaging	If a preoperative cone-beam computed tomographic scan has not been obtained, CBCT is indicated for identification and localization of calcified canals,^8^ canal branching, perforations, or canal obstruction.	CBCT aids in identification of the spatial location of extensively obliterated canals and assists in guided endodontics.^51^
**Posttreatment Evaluation (Follow-Up)**		
Postoperative imaging	Periapical radiographs are indicated for postoperative evaluation^8^ unless there is evidence of persistent disease, when small field-of-view CBCT should be considered to identify possible etiology and optimal treatment plan.	NA^§^
Diagnosing vertical root fractures and crown-to-root fractures	Periapical radiographs or CBCT may be indicated. CBCT is the imaging modality of choice when clinical examination and intraoral radiography are inconclusive.^8,51^	Periapical radiographs are generally better in endodontically-treated teeth; CBCT in non—endodontically-treated teeth.^54^ However, CBCT is useful in the detection of patterns of periradicular bone changes indicative of root fractures, when clinical examination and 2D imaging modalities are not conclusive.^51,59^
Evaluating endodontic treatment complications	CBCT is indicated for assessment of perforations,^8,51^ root resorption that may be amenable to surgical or nonsurgical retreatment,^51^ or other complications.^8^	NA
Nonsurgical retreatment	CBCT is indicated to localize root apexes and locate adjacent structures, canal obstruction, thickness of remaining dentin, major voids, or irregularities in obturation.^8,51^	NA
Surgical retreatment	CBCT was found to have higher accuracy than 2D imaging with respect to definitive assessment of surgical endodontic treatment outcomes.^60^	NA

* 2D: 2-Dimensional.

† CBCT: Cone-beam computed tomography.

‡ 2024 American Dental Association Council on Scientific Affairs Recommendation 3.2.1: “CBCT imaging should not be used routinely. CBCT examinations shall not be used as the primary or initial imaging modality when a lower dose alternative is adequate for diagnosis and treatment planning.”^2^

§ NA: Not applicable.

**Table 2.** Initial imaging recommendations based on type of encounter.

TYPE OF ENCOUNTER (NEW OR RECALL VISIT)	CHILD WITH PRIMARY DENTITION (BEFORE ERUPTION OF FIRST PERMANENT TOOTH)	CHILD WITH TRANSITIONAL DENTITION (AFTER ERUPTION OF FIRST PERMANENT TOOTH)	ADOLESCENT WITH PERMANENT DENTITION (BEFORE ERUPTION OF THIRD MOLARS)	ADULT, DENTATE OR PARTIALLY EDENTULOUS
**New Patient Evaluation**	Individualized radiographic examination with periapical or occlusal views or posterior bite-wing radiographs if proximal surfaces cannot be visualized or probed. Patients without evidence of disease and with open proximal contacts may not require a radiographic examination.	Individualized radiographic examination consisting of posterior bite-wing radiographs with panoramic examination or posterior bite-wing radiographs and selected periapical images.	Individualized radiographic examination consisting of posterior bite-wing radiographs with panoramic examination or posterior bite-wing radiographs and selected periapical images. A full-mouth intraoral radiographic examination is preferred when the patient has clinical evidence of generalized oral disease or a history of extensive dental treatment.	Individualized radiographic examination consisting of posterior bite-wing radiographs with panoramic examination or posterior bite-wing radiographs and selected periapical images. A full-mouth intraoral radiographic examination is preferred when the patient has clinical evidence of generalized oral disease or a history of extensive dental treatment.
**Recall Visit, Patient With No Clinical Caries and Not at Increased Risk of Developing Caries**	Posterior bite-wing examination at 12- to 24-m intervals if proximal surfaces cannot be examined visually or with a probe.	Posterior bite-wing examination at 12- to 24-m intervals if proximal surfaces cannot be examined visually or with a probe.	Posterior bite-wing examination at 18- to 36-mo intervals.	Posterior bite-wing examination at 24- to 36-mo intervals.
**Recall Visit, Patient With Clinical Caries or at Increased Risk of Developing Caries**	Posterior bite-wing examination at 6- to 12-mo intervals if proximal surfaces cannot be examined visually or with a probe.	Posterior bite-wing examination at 6- to 12-mo intervals if proximal surfaces cannot be examined visually or with a probe.	Posterior bite-wing examination at 6- to 12-mo intervals if proximal surfaces cannot be examined visually or with a probe.	Posterior bite-wing examination at 6- to 18-mo intervals.
**Recall Visit, Patient With Periodontal Disease**	Clinical judgment as to the need for and type of radiographic images for evaluation of periodontal disease. Imaging may consist of, but is not limited to, selected bite-wing or periapical images of areas where periodontal disease (other than nonspecific gingivitis) can be found clinically.	Clinical judgment as to the need for and type of radiographic images for evaluation of periodontal disease. Imaging may consist of, but is not limited to, selected bite-wing or periapical images of areas where periodontal disease (other than nonspecific gingivitis) can be found clinically.	Clinical judgment as to the need for and type of radiographic images for evaluation of periodontal disease. Imaging may consist of, but is not limited to, selected bite-wing or periapical images of areas where periodontal disease (other than nonspecific gingivitis) can be found clinically.	Clinical judgment as to the need for and type of radiographic images for evaluation of periodontal disease. Imaging may consist of, but is not limited to, selected bite-wing or periapical images of areas where periodontal disease (other than nonspecific gingivitis) can be found clinically.

## Box 1 Radiography recommendations

**Table:**

**1.0**	**General Recommendations**
1.1	Adhere to professional guidance and federal, state, or local laws and public health guidance relevant to patient safety and operator protection, education, and training; dose optimization and exposure settings, and quality assurance and quality control regarding any radiographic or CBCT* imaging in dentistry.^1,2,7^
1.2	Radiographic screening shall not be performed before the clinical examination. The decision to obtain a radiograph or CBCT scan shall be made on the basis of the patient’s medical and dental histories, clinical examination, disease risk assessment, presence of a clinical condition, and previously obtained radiographic imaging. The benefits to the diagnosis and treatment planning must outweigh the potential risks from exposure to radiation, especially in the case of a child or young adult.^2,7^
1.3	All radiographs should be examined for any evidence of caries, calculus, alveolar bone loss, developmental or acquired anomalies, and other pathoses, in accordance with professional guidelines.^2,7,8^
1.4	Intraoral radiography is useful for the evaluation of dentoalveolar trauma. If the area of interest extends beyond the dentoalveolar complex, extraoral imaging may be indicated.^2,7,9,10^
1.5	Dental staff members and operators of imaging equipment shall be trained in appropriate techniques and patient positioning and remove objects that could affect the diagnostic quality.^1,2,7^
1.6	Clinicians, dental staff members, and operators of imaging equipment must be knowledgeable of the radiation risks from radiographic and CBCT imaging and able to communicate these risks to their patients.^1,2,7^
1.7	CBCT is not indicated for caries detection.^11^

* CBCT: Cone-beam computed tomography.

## Box 2 Indications.

**Table:**

**1.0**	**Imaging Recommendations for Caries Indications**
1.1	CBCT* is not indicated for caries detection.^12–15^
1.2	For anterior proximal caries that are not clinically visible, periapical radiographs are indicated.^3^
1.3	For posterior proximal caries that are not clinically visible, bite-wing radiographs are indicated.^3,12^
1.4	For diagnosis and depth estimation of proximal caries on clinically visible surfaces, periapical or bite-wing radiographs may be indicated.^3,12^
1.5	For occlusal caries, periapical or bite-wing radiographs may be indicated.^3,12^
1.6	For root caries, periapical or bite-wing radiographs may be indicated.^3,12,16^
1.7	For diagnosis and depth estimation of smooth surface caries, visual examination is preferred over radiographic imaging in teeth with no clinical signs or symptoms associated with pulpitis or apical periodontitis.^3^
**2.0**	**Imaging Recommendations for Periodontal Disease**
2.1	The frequency of radiographic examination for the patient with periodontal disease is dependent on the clinical findings and treatment response.^3,17^
2.2	Radiographic documentation of the disease sites before periodontal treatment is important for diagnosis, baseline documentation, and treatment planning.^3,17^
2.3	Radiographic evidence of the presence and severity of periodontal bone loss does not indicate active disease.^3,17^
2.4	For evaluating periodontal structures after comprehensive clinical examination, 2D^†^ full-mouth radiographic series, including vertical bite-wing radiographs as necessary, is recommended.^3,17^
2.5	Management of periodontal disease, in general, does not warrant use of CBCT, although CBCT may be indicated for treatment planning of complex cases.^3,17^
2.6	For patients with history of, or with active, periodontal disease, vertical bite-wing radiographs are recommended to assess bone levels in the permanent dentition.^3,17^
**3.0**	**Imaging Recommendations for Orthodontic Indications**
3.1	For monitoring of tooth eruption before initiation of orthodontic treatment and assessment of root alignment during treatment, panoramic radiographs should be used as the initial imaging modality.^18–20^
3.2	For assessment of the severity of a class II or class III malocclusion with or without a vertical component, lateral cephalogram may be used.^18–21^
3.3	To assess root morphology, root blunting or resorption, and periapical lesions, periapical radiographs are most appropriate.^18–23^
3.4	For assessing facial asymmetry, low-dose CBCT is recommended. When low-dose CBCT is not available, posteroanterior cephalometric radiograph may be used.^19,20,24^
3.5	For interradicular mini-implants, CBCT can aid in optimal site selection and has been found to improve the mini-implant success rate compared with 2D radiographs alone. However, the benefits of CBCT should be weighed against the increased radiation dose on a case-by-case basis.^20,25^
3.6	For clinical indications, such as suspected pathosis, delayed eruption, or orthodontic concerns, panoramic radiography is recommended for assessment and treatment planning. However, panoramic imaging should be avoided unless justified by substantial changes in clinical status or developmental stage.^26–32^
**4.0**	**Imaging Recommendations for Third Molars, Supernumerary Teeth, and Supplemental Teeth**
4.1	Routine radiographic screening for third molars, supernumerary teeth, and supplemental teeth without a clinical indication is not recommended.^3,4^
4.2	When there is a clinical indication for radiographic evaluation of third molars, supernumerary teeth, and supplemental teeth, panoramic radiography is recommended for assessment and treatment planning.^26–33^
4.3	The frequency of panoramic radiography for third molars, supernumerary teeth, and supplemental teeth should be determined on the basis of stage of dental development and clinical need.^3,26–29,33^
4.4	If panoramic radiography indicates an increased risk of experiencing an inferior alveolar nerve injury (ie, apex of the root is below the canal, darkening of the roots, loss of the cortical outline of the canal, or diversion of the canal), CBCT imaging should be considered.^30,31,33^
4.5	CBCT should only be used if radiographic findings will affect risk assessment or treatment decisions.^29–32^
**5.0**	**Imaging Recommendations for Head and Neck Lesions**
5.1	The prescription, type, and frequency of radiographic examination for head and neck pathoses should be determined on the basis of medical and dental histories clinical findings, potential diagnoses, and existing imaging.^2,3^
5.2	An individualized approach should be followed when selecting the most appropriate imaging modality for the evaluation of jaw lesions, given the broad spectrum of diseases.^2,3,34^
5.3	The oral and maxillofacial pathology, oral medicine, and orofacial pain disciplines use various imaging modalities, depending on clinical parameters, such as manifestation, history, symptomatology, examination findings, laboratory or test results, and therapeutics as relevant to each case. Such imaging modalities include, but are not limited to, 2D radiography, CBCT, MDCT,^‡^ positron emission tomography, MRI,^§^ magnetic resonance angiography, and ultrasonography, all of which must be determined using a benefit-to-risk decision-making process.
5.4	If pathosis is suspected or identified on radiographic images, then appropriate referral to a specialty provider type may be warranted before additional imaging.
**6.0**	**Imaging Recommendations for TMD**^¶^ **and Orofacial Pain**
6.1	For initial imaging of patients with suspected TMJ^#^ disorders, panoramic radiography may be considered to rule out gross osseous abnormalities. However, due to low sensitivity, it may not be sufficient for definitive TMJ diagnosis.^35^
6.2	CBCT is the preferred imaging modality to assess bony components of the TMJ, such as degenerative joint disease, idiopathic condylar resorption, systemic arthritides, or developmental anomalies.^35,36^
6.3	When there is a suspected condylar fracture or trauma to the TMJ region, MDCT or CBCT is preferred to characterize fracture location, displacement, and associated hard-tissue injury,^35^ such as ankyloses. MRI is preferred for suspected soft-tissue trauma, such as the presence of adhesions and for the detection of hermarthrosis.^37–39^
6.4	MRI should be used for definitive assessment of soft-tissue TMJ pathology, including disk displacement, joint effusion, and inflammatory changes. T1- or proton density weighted MRI is recommended for disk morphology and position and T2-weighted MRI is recommended for joint effusion.^35,40^
6.5	TMJ internal derangements are common and do not require imaging unless functionally limiting. Open- and closed-mouth MRI imaging is recommended for evaluating TMJ disk displacements.^35,39^
6.6	Imaging should only be used when clinical findings suggest a need for further investigation or treatment planning and not as a routine part of TMD diagnosis.^39,41^
6.7	When orofacial pain is suspected to be of non-TMD origin and nonodontogenic origin, such as neurovascular, neuropathic, neoplastic, systemic infectious, or other secondary causes, appropriate referral to medical or dental specialists is recommended. Advanced imaging modalities, such as MRI, magnetic resonance angiography, MDCT, or positron emission tomography may be indicated on the basis of the suspected etiology to support accurate diagnosis and management.^39,41,42^
**7.0**	**Imaging Recommendations for Dental Implants**
**7.1**	**Initial Consultation**
7.1.1	Initial assessment before dental implants, panoramic radiography may be performed. However, for presurgical planning and placement of dental implants, CBCT is recommended (see section 6.2).^43–45^
**7.2**	**Applications of CBCT in Implant Therapy**
7.2.1	When results of clinical examination indicate bone grafting or reconstruction will be needed, 3-dimensional assessment of the implant site is recommended.^43–45^
7.2.2	The relation of relevant anatomic structures to the implant site should be assessed using CBCT.^43–45^
7.2.3	3-Dimensional assessment of bone volume of the edentulous sites receiving dental implants is recommended.^43^
7.2.4	The maxillary sinus and alveolar ridge before augmentation procedure should be assessed with CBCT.^43–45^
7.2.5	The autogenous bone donor site should be evaluated with CBCT.^43–45^
7.2.6	Fabrication of surgical guides that are static or those used during dynamic navigation implant placement necessitates the need for CBCT.^43–45^
7.2.7	Implant sites that have been augmented previously should be assessed using CBCT.^43–45^
7.2.8	CBCT should be used to assess complications in implants placed previously.^43–46^
**7.3**	**Restoration**
7.3.1	At the time of restoration delivery, 2D intraoral radiographs (eg, bite-wing) perpendicular to the implant should be obtained to provide baseline peri-implant bone level information for long-term follow-up.^45^
**7.4**	**Maintenance**
7.4.1	Periodic 2D intraoral radiographic evaluation of implants should be performed as an adjunct to recall visit or maintenance examinations on the basis of the clinical judgment of the dental care professional.^3,45^
7.4.2	To assess peri-implant bone, 2D intraoral radiography is the imaging modality of choice.^43,45^
7.4.3	To assess complications likely due to improper anatomic location of the implant, CBCT is the imaging modality of choice.^43,45–47^
**8.0**	**Applications of CBCT in Autotransplantation**
8.1	To assess the integrity of the donor tooth and the recipient site, CBCT is recommended.^48–50^
8.2	For the fabrication of the replica donor tooth to be used for try-in, CBCT is recommended.^48–50^
8.3	Survival of the autotransplanted tooth should be assessed using 2D imaging.^3^

* CBCT: Cone-beam computed tomography.

† 2D: 2-Dimensional.

‡ MDCT: Multidetector computed tomography.

§ MRI: Magnetic resonance imaging.

¶ TMD: Temporomandibular disorder.

# TMJ: Temporomandibular joint.

## Box 3 Pediatric recommendations.

**Table:**

**1.0**	**General Recommendations for Pediatric Dentistry**
1.1	The frequency of radiographic examination for the patient in the primary, mixed, or early adolescent dentition is dependent on the medical history (including medications and surgical and dental histories), clinical examination, caries and periodontal risk assessment, and trauma experience assessment.^3,27^
1.2	Follow-up radiographic examinations after the initial radiographic examination should be based on caries and periodontal disease risk, eruption of teeth, dental and medical histories, and clinical findings.^3,11,27^
1.3	Radiographic examination after dental trauma is indicated in addition to a thorough clinical examination in accordance with the International Association of Dental Traumatology guidelines.^9,10^
1.4	Children and young adults are more susceptible to the effects of radiation, so radiographs should be ordered judiciously.^2,3,11,61^
1.5	With dose-reduction efforts recommended by the American Dental Association and American Academy of Oral and Maxillofacial Radiology (ie, selection criteria, collimation, and optimization of exposure settings), radiation doses from dentomaxillofacial imaging carry negligible risk; therefore, routine use of lead shielding for pediatric patients is not indicated.^2,7^
**2.0**	**Intraoral Radiography–Specific Recommendations**
2.1	For patients in the primary, mixed, or early adolescent dentition with severe gingival inflammation as a result of systemic conditions, maxillary and mandibular occlusal or periapical and bite-wing radiographs shall be obtained along with mobility testing as part of the initial assessment in lieu of periodontal probing of hemorrhagic gingiva.^3,27^
2.1.1	For the assessment of bone levels in adolescents with periodontal disease, in the permanent dentition, vertical bite-wing radiographs should be used.
**3.0**	**Extraoral Radiography–Specific Recommendations**
3.1	An initial panoramic radiograph should only be obtained after complete eruption of permanent first molars and all mandibular incisors unless needed earlier to assess oral pathosis.
3.2	Extraoral bite-wing radiographs should be obtained judiciously because they provide a radiation dose that is 3 times higher than intraoral bite-wing radiographs and may have lower diagnostic quality. Extraoral bite-wing radiographs may be used for children with special health care needs or who are unable to tolerate intraoral radiography. They should only be considered after clinical examination and assessment for caries and periodontal disease risk, trauma experience, eruption deviations, and third molars.^2,3,12^
**4.0**	**CBCT** * **-Specific Recommendations**
4.1	The evidence does not support CBCT for caries detection.^11,13–15^
4.2	When 2-dimensional imaging does not provide adequate information, such as during suspected pathosis, trauma, or localization of impacted teeth, CBCT may be considered.^11^
4.3	The patient’s ability to follow instructions and hold still should be considered when ordering radiographs, especially for longer exposure time modalities, like panoramic radiography or CBCT.^11^

* CBCT: Cone-beam computed tomography.
