# Supplementary material for: American Dental Association and American Academy of Oral and Maxillofacial Radiology patient selection for dental radiography and cone-beam computed tomography
Source: Oral Surg Oral Med Oral Pathol Oral Radiol. Author manuscript; Available in PMC 2026 Jun 7. (PMC13242894; doi:10.1016/j.oooo.2025.11.013)
Supplement: Appendix A and B [file NIHMS2182358-supplement-Appendix_A_and_B.docx]

Appendix A:
Indication / recall search strategies
Ovid MEDLINE search strategy

| 1 | exp Radiography, Dental/ |
| --- | --- |
| 2 | ((radiograph$ or x-ray$ or radiation or radiology or radiolucency or radiopacity or radiopaque or radiolucent or imaging or bitewing or CBCT or "Cone-beam CT" or "cone beam computed tomography" or "Computerized tomography" or panoramic or orthopantomograph$) adj5 (dent$ or tooth or teeth or orthodont$ or mouth or maxillofacial or endodont$ or periodont$ or root or maxillary or gingiv$ or intraoral or periapical or alveolar or molar or premolar or cuspid or incisor or canine or temporomandibular or furcation or 'intrabony defect' or 'dental caries' or 'carious lesion')).ab,kw,ti. |
| 3 | 1 or 2 |
| 4 | exp Time Factors/ |
| 5 | (frequency or frequent or frequently or rate or often or recall or periodically or "prescribing practices" or "time interval" or "time intervals" or timing or routine or ALADA or "as low as reasonably achievable" or ALARA).ab,kw,ti. |
| 6 | 4 or 5 |
| 7 | (indication$ or indicated or prescribe or prescribed or prescribing or prescription or "selection criteria").ab,kw,ti. |
| 8 | 6 or 7 |
| 9 | 3 and 8 |
| 10 | Meta-Analysis as Topic/ |
| 11 | meta analy$.tw. |
| 12 | metaanaly$.tw. |
| 13 | Meta-Analysis/ |
| 14 | (systematic adj (review$1 or overview$1)).tw. |
| 15 | exp "Review Literature as Topic"/ |
| 16 | review.pt. |
| 17 | 10 or 11 or 12 or 13 or 14 or 15 or 16 |
| 18 | cochrane.ab. |
| 19 | embase.ab. |
| 20 | (psychlit or psyclit).ab. |
| 21 | (psychinfo or psycinfo).ab. |
| 22 | (cinahl or cinhal).ab. |
| 23 | science citation index.ab. |
| 24 | bids.ab. |
| 25 | cancerlit.ab. |
| 26 | 18 or 19 or 20 or 21 or 22 or 23 or 24 or 25 |
| 27 | reference list$.ab. |
| 28 | bibliograph$.ab. |
| 29 | hand-search$.ab. |
| 30 | relevant journals.ab. |
| 31 | manual search$.ab. |
| 32 | 27 or 28 or 29 or 30 or 31 |
| 33 | exp guideline/ |
| 34 | (guideline or guidelines).ab,kw,ot,ti. |
| 35 | ('consensus statement' or 'consensus statements').ab,kw,ot,ti. |
| 36 | 33 or 34 or 35 |
| 37 | selection criteria.ab. |
| 38 | data extraction.ab. |
| 39 | 37 or 38 |
| 40 | "Review"/ |
| 41 | 39 and 40 |
| 42 | Comment/ |
| 43 | Letter/ |
| 44 | Editorial/ |
| 45 | exp Animals/ |
| 46 | exp Humans/ |
| 47 | 45 and 46 |
| 48 | 45 not 47 |
| 49 | 42 or 43 or 44 or 48 |
| 50 | 17 or 26 or 32 or 36 or 41 |
| 51 | 50 not 49 |
| 52 | 9 and 51 |

Embase search strategy

| 1 | 'dental x ray system'/exp |
| --- | --- |
| 2 | 'dental radiology'/exp |
| 3 | ((dent* OR tooth OR teeth OR orthodont* OR mouth OR maxillofacial OR endodont* OR periodont* OR root OR maxillary OR gingiv* OR intraoral OR periapical OR alveolar OR molar OR premolar OR cuspid OR incisor OR canine OR temporomandibular OR furcation OR 'intrabony defect' OR 'dental caries' OR 'carious lesion') NEAR/5 (radiograph* OR 'x ray*' OR radiation OR radiology OR radiolucency OR radiopacity OR radiopaque OR radiolucent OR imaging OR bitewing OR cbct OR 'cone-beam ct' OR 'cone beam computed tomography' OR 'computerized tomography' OR panoramic OR orthopantomograph*)):ab,ti,kw |
| 4 | #1 OR #2 OR #3 |
| 5 | 'time factor'/exp |
| 6 | frequency:ti,ab,kw OR frequent:ti,ab,kw OR frequently:ti,ab,kw OR rate:ti,ab,kw OR often:ti,ab,kw OR recall:ti,ab,kw OR periodically:ti,ab,kw OR 'prescribing practices':ti,ab,kw OR 'time interval':ti,ab,kw OR 'time intervals':ti,ab,kw OR timing:ti,ab,kw OR routine:ti,ab,kw OR alada:ti,ab,kw OR 'as low as reasonably achievable':ti,ab,kw OR alara:ti,ab,kw |
| 7 | #5 OR #6 |
| 8 | indication*:ti,ab,kw OR indicated:ti,ab,kw OR prescribe:ti,ab,kw OR prescribed:ti,ab,kw OR prescribing:ti,ab,kw OR prescription:ti,ab,kw OR 'selection criteria':ti,ab,kw |
| 9 | 'meta analysis'/exp OR 'review'/exp OR 'review':it OR 'systematic review'/exp OR 'systematic review':it |
| 10 | (meta NEXT/1 analy*) OR metaanalys* |
| 11 | systematic* NEAR/5 (review* OR overview*) |
| 12 | #9 OR #10 OR #11 |
| 13 | guideline:ti,ab,kw OR guidelines:ti,ab,kw |
| 14 | 'practice guideline'/exp |
| 15 | 'consensus statement':ti,ab,kw OR 'consensus statements':ti,ab,kw |
| 16 | #13 OR #14 OR #15 |
| 17 | 'cancerlit':ab |
| 18 | 'cochrane':ab |
| 19 | 'embase':ab |
| 20 | 'psychlit':ab OR 'psyclit':ab |
| 21 | 'psychinfo':ab OR 'psycinfo':ab |
| 22 | 'cinahl':ab OR 'cinhal':ab |
| 23 | 'science citation index':ab |
| 24 | 'bids':ab |
| 25 | #17 OR #18 OR #19 OR #20 OR #21 OR #22 OR #23 OR #24 |
| 26 | 'reference lists':ab |
| 27 | 'bibliograph*':ab |
| 28 | 'hand-search*':ab |
| 29 | 'manual search*':ab |
| 30 | 'relevant journals':ab |
| 31 | #26 OR #27 OR #28 OR #29 OR #30 |
| 32 | 'letter':it |
| 33 | 'editorial':it |
| 34 | 'animal'/exp |
| 35 | 'human'/exp |
| 36 | #34 NOT (#34 AND #35) |
| 37 | #32 OR #33 OR #36 |
| 38 | #12 OR #16 OR #25 OR #31 |
| 39 | #38 NOT #37 |
| 40 | #7 OR #8 |
| 41 | #4 AND #40 |
| 42 | #39 AND #41 |

Cochrane Database of Systematic Reviews search strategy

| 1 | MeSH descriptor: [Radiography, Dental] explode all trees |
| --- | --- |
| 2 | ((radiograph* OR x-ray* OR radiation OR radiology OR radiolucency OR radiopacity OR radiopaque OR radiolucent OR imaging OR bitewing OR CBCT OR "Cone-beam CT" OR "cone beam computed tomography" OR "Computerized tomography" OR panoramic OR orthopantomograph*) NEAR/5 (dent* OR tooth OR teeth OR orthodont* OR mouth OR maxillofacial OR endodont* OR periodont* OR root OR maxillary OR gingiv* OR intraoral OR periapical OR alveolar OR molar OR premolar OR cuspid OR incisor OR canine OR temporomandibular OR furcation OR 'intrabony defect' OR 'dental caries' OR 'carious lesion')):ti,ab,kw (Word variations have been searched) |
| 3 | #1 OR #2 |
| 4 | MeSH descriptor: [Time Factors] explode all trees |
| 5 | (frequency OR frequent OR frequently OR rate OR often OR recall OR periodically OR "prescribing practices" OR "time interval" OR "time intervals" OR timing OR routine OR ALADA OR "as low as reasonably achievable" OR ALARA):ti,ab,kw (Word variations have been searched) |
| 6 | #4 OR #5 |
| 7 | (indication* OR indicated OR prescribe OR prescribed OR prescribing OR prescription OR "selection criteria"):ti,ab,kw |
| 8 | #6 OR #7 |

PRISMA FLOW DIAGRAM

**Identification of studies via databases and registers**

Records removed *before screening*:

Duplicate records removed (n = 0)

Records identified from*:

Databases (n = 810)

**Identification**

Records screened

(n = 810)

Records excluded**

(n = 540)

Reports sought for retrieval

(n = 270)

**Screening**

Reports assessed for eligibility

(n = 270)

Reports excluded, with reasons: (n = 196)

Studies proposed for inclusion

(n = 74)

**Included**

*Consider, if feasible to do so, reporting the number of records identified from each database or register searched (rather than the total number across all databases/registers).

**If automation tools were used, indicate how many records were excluded by a human and how many were excluded by automation tools.

Source: Page MJ, et al. BMJ 2021;372:n71. doi: 10.1136/bmj.n71.

This work is licensed under CC BY 4.0. To view a copy of this license, visit <https://creativecommons.org/licenses/by/4.0/>

Appendix B:

**9 Clinical Questions for Radiography Update to the 2012 Recommendations**

1. What radiologic evaluation is recommended during patient recall appointment for individuals with clinical caries and/or in professional opinion are at increased risk for caries?
2. What radiologic evaluation is recommended during patient recall appointment for individuals with no caries and/or in professional opinion do not seem to be at increased risk for caries?
3. What radiologic evaluation is recommended for new patients?
4. What radiologic evaluation is recommended during patient recall appointment for individuals with periodontal disease?
5. What radiologic evaluation is recommended assess dentofacial development?
6. What are positive clinical signs/symptoms for clinical situations in which radiologic evaluation may be indicated?
7. Which radiologic evaluations are appropriate for planning, intraoperative, and follow-up evaluation for implants?
8. Which radiologic evaluations are appropriate for diagnosis, intraoperative and follow-up evaluation for endodontic lesions?
9. What radiologic evaluation is recommended for assessment of dental trauma?
