# Supplementary figures and images for: American Dental Association and American Academy of Oral and Maxillofacial Radiology patient selection for dental radiography and cone-beam computed tomography
Source: Oral Surg Oral Med Oral Pathol Oral Radiol. Author manuscript; Available in PMC 2026 Jun 7. (PMC13242894; doi:10.1016/j.oooo.2025.11.013)

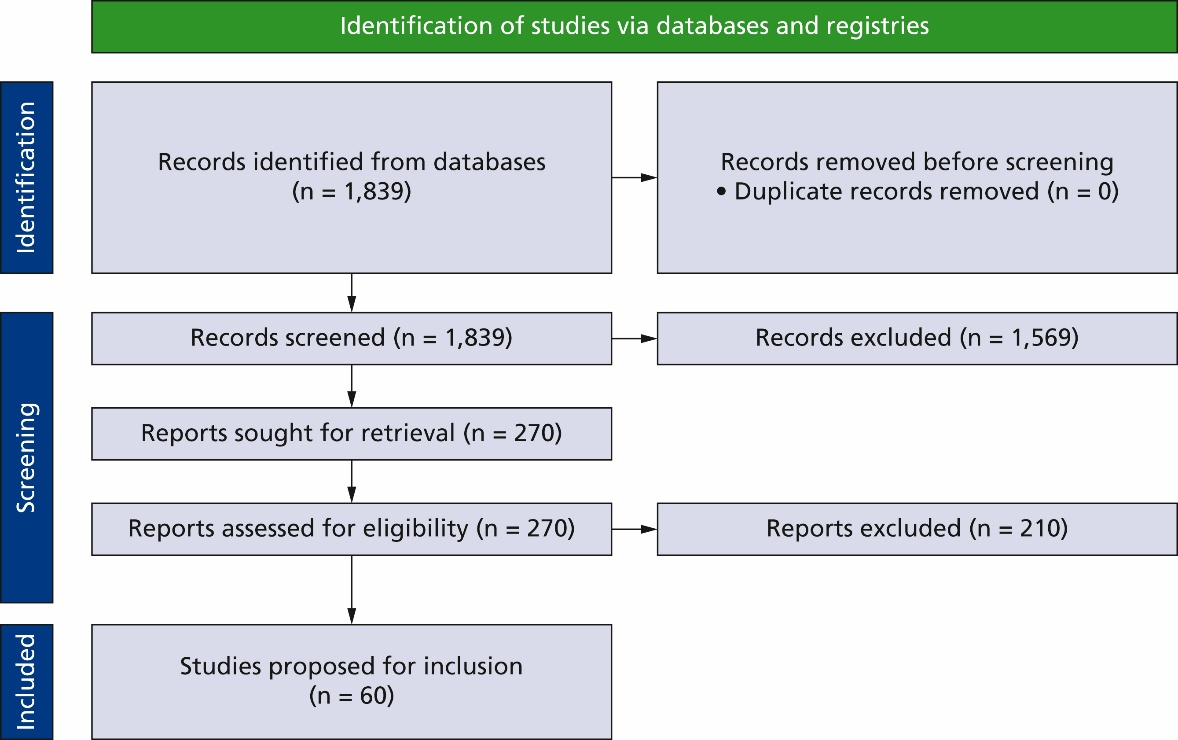


eFigure. Preferred Reporting Items for Systematic Reviews and Meta-Analyses flow diagram. 73

Supplement: efigure [file NIHMS2182358-supplement-efigure.docx]
